# Complete Genome Sequences of 10 Phages Lytic against Multidrug-Resistant Pseudomonas aeruginosa

**DOI:** 10.1128/MRA.00503-20

**Published:** 2020-07-16

**Authors:** Jason Farlow, Helen R. Freyberger, Yunxiu He, Amanda M. Ward, Wiriya Rutvisuttinunt, Tao Li, Ross Campbell, Anna C. Jacobs, Mikeljon P. Nikolich, Andrey A. Filippov

**Affiliations:** a Wound Infections Department, Bacterial Diseases Branch, Walter Reed Army Institute of Research, Silver Spring, Maryland, USA; b Viral Diseases Branch, Walter Reed Army Institute of Research, Silver Spring, Maryland, USA; c Farlow Scientific Consulting, LLC, Lewiston, Utah, USA; d The Geneva Foundation, Fort Detrick, Maryland, USA; Loyola University Chicago

## Abstract

We report the genome sequences of 10 Pseudomonas aeruginosa phages studied for their potential for formulation of a therapeutic cocktail; they represent the families *Myoviridae*, *Podoviridae*, and *Siphoviridae*. Genome sizes ranged from 43,299 to 88,728 nucleotides, with G+C contents of 52.1% to 62.2%. The genomes contained 68 to 168 coding sequences.

## ANNOUNCEMENT

In the context of limited success of antibiotics, phages are promising alternative antibacterials. Phages have demonstrated therapeutic efficacy against Pseudomonas aeruginosa infections in animals (1) and humans (1–3). Since P. aeruginosa phages have narrow host ranges (4, 5), phage cocktails are required to cover most clinical isolates (6). We are developing a phage cocktail that is active against the majority of multidrug-resistant (MDR) P. aeruginosa isolates from traumatic and burn wounds. Here, we report the whole-genome sequences of 10 P. aeruginosa phages isolated from sewage (Table 1). Each phage lysed 23 to 58% of 156 diverse MDR isolates. The phages were complementary to each other (their mixes showed broader activity than single phages).

**TABLE 1:** Genomic attributes of the 10 P. aeruginosa phages

Phage name	Family	Genus	Genome length (bp)	G+C content (%)	No. of protein-coding genes	Genome coverage (×)	No. of raw reads	GenBank accession no.	SRA accession no.
EPa1	*Podoviridae*	*Bruynoghevirus*	45,230	52.1	76	158.1	308,634	MT108723	SAMN15311669
EPa2	*Podoviridae*	*Bruynoghevirus*	43,299	52.3	68	757.2	302,307	MT108724	SAMN15311670
EPa5	*Siphoviridae*	*Abidjanvirus*	63,969	62.2	91	1,672.2	534,271	MT108725	SAMN15311671
EPa6	*Myoviridae*	*Pbunavirus*	66,031	55.1	95	70.4	202,626	MT108726	SAMN15311672
EPa11	*Myoviridae*	*Pbunavirus*	66,800	55.7	95	1,004.6	272,627	MT108727	SAMN15311673
EPa15	*Myoviridae*	*Pbunavirus*	66,002	55.6	95	1,197.8	479,511	MT413450	SAMN15311674
EPa17	*Myoviridae*	*Nankokuvirus*	88,600	54.8	164	2,099.6	671,404	MT108728	SAMN15311675
EPa22	*Myoviridae*	*Pbunavirus*	65,897	55.4	98	1,556.5	457,227	MT108729	SAMN15311676
EPa24	*Myoviridae*	*Nankokuvirus*	88,728	54.8	168	4,728.4	1,577,519	MT108730	SAMN15311677
EPa43	*Siphoviridae*	*Abidjanvirus*	64,323	62.0	97	2,003.0	398,955	MT108731	SAMN15311678

The phages were isolated from sewage collected in Washington, DC. P. aeruginosa strain PAO1 was used for enrichment. Phages were purified by three rounds of single-plaque isolation, propagated on strain PAO1 in broth, and concentrated by high-speed centrifugation as described previously (7). Host RNA and DNA were removed from lysates with RNase A and DNase, respectively, and phage DNA was isolated using proteinase K and SDS treatment followed by phenol-chloroform extraction, overnight precipitation with ethanol at −20°C, centrifugation, and resuspension in nuclease-free water (7). Phage DNA was sequenced using a Nextera XT DNA library preparation kit (Illumina, San Diego, CA). Libraries were validated and quantified using a TapeStation D5000 kit (Agilent Technologies, Inc., Santa Clara, CA) and an Invitrogen Qubit double-stranded DNA (dsDNA) broad-range (BR) assay kit (Thermo Fisher Scientific, Waltham, MA), respectively, purified with AMPure XP beads (Beckman Coulter Diagnostics, Brea, CA), and sequenced using a 600-cycle MiSeq reagent kit v3 on an Illumina MiSeq system, producing 300-bp paired-end reads. FastQC v0.11.5 (https://www.bioinformatics.babraham.ac.uk/projects/fastqc) was used for read quality control. Raw reads listed in Table 1 for each phage were subsequently trimmed using default parameters in Geneious Prime v2019.2.3 and were subjected to *de novo* assembly using default parameters in PATRIC (8). Phage genome annotations were carried out using the RAST server (9). Nucleic acid sequence similarity searches were performed using default parameters in BLASTn (10).

Phage genome sizes ranged from 43,299 to 88,728 nucleotides, with G+C contents of 52.1% to 62.2% (Table 1). The genomes contained 68 to 168 coding sequences. Phages EPa1 and EPa2 (family *Podoviridae,* genus *Bruynoghevirus*) were closely related to lytic phage LUZ24 (GenBank accession number AM910650.1) (11), based on BLASTn sequence comparisons. The phage genomes lacked significant nucleic acid sequence similarity to genes encoding integrases, recombinases, transposases, excisionases, and repressors of the lytic cycle. Therefore, EPa1 and EPa2 appear to be obligatorily lytic. Six *Myoviridae* phages, namely, EPa6, EPa11, EPa15, and EPa22 (genus *Pbunavirus*) and EPa17 and EPa24 (genus *Nankokuvirus*), also lacked genes typical of temperate phages, suggesting that they are strictly virulent, similar to other genus *Pbunavirus* (12) and *Nankokuvirus* (13) members. BLASTn and BLASTp analyses found no significant similarity in any of the eight phages to bacterial DNA and proteins, including drug resistance and pathogenicity determinants. Our data suggest that the eight phages are promising therapeutic candidates.

However, *Siphoviridae* phages EPa5 and EPa43, with high lytic potential, encoded putative proteins described as an integrase and a repressor in genome annotations of other phages, including Ab18, Ab19, Ab20, and Ab21, belonging to the genus *Abidjanvirus* (open reading frame 22 [ORF22] and ORF21 in the Ab18 genome [GenBank accession number LN610577]) (14). Subsequent inspection revealed only primase-related domains and a lack of integrase-associated domains in the ORF22 product in EPa5, EPa43, and related phages. The ORF21 homolog contained an HTH_XRE domain, which is common in phages and has been associated with transcriptional antirepressor and repressor activities but remains largely uncharacterized. BLASTn and BLASTp searches for phages EPa5 and EPa43 did not identify any significant similarity to bacterial genes or proteins. Additional analysis is required to consider these two phages safe for therapeutic purposes.

### Data availability.

The 10 complete phage genome sequences were deposited in GenBank and the NCBI Sequence Read Archive (SRA) under the accession numbers listed in Table 1.
